# A novel prototype-based segmentation requiring only five training cases applied to MR angiography

**DOI:** 10.1186/1532-429X-11-S1-P109

**Published:** 2009-01-28

**Authors:** Jane Sjögren, Martin Ugander, Håkan Arheden, Einar Heiberg

**Affiliations:** 1 Dept. of Clinical Physiology, Cardiac MR Group, Lund, Sweden; 2 Lund University Hospital, Lund, Sweden

**Keywords:** Segmentation Algorithm, Steady State Free Precession, Absolute Distance, Segmentation Error, Volumetric Error

## Introduction

Image segmentation is an important pre-processing step and a prerequisite for visualization and quantification in medical imaging. In order to obtain accurate segmentation in MRI a priori information often needs to be used. Existing methods often construct a statistical model of the object to be segmented and this typically requires 50 – 100 manually segmented cases.

## Purpose

To develop and assess the accuracy of a novel prototype-based segmentation method where few cases, typically five, are needed to introduce the required a priori information.

## Methods

Ten healthy volunteers underwent contrast enhanced MR imaging of the aorta at 1.5 T (Philips). Imaging employed a steady state free precession sequence, resolution 1.6 × 1.6 × 1 mm. The main idea behind the novel prototype-based segmentation algorithm is to use spatial a priori information to restrict the segmentation rather than to govern what to include. A priori information was extracted from five of the cases (training set) and stored into a prototype. Manual delineation of the aorta was performed in all ten cases and four anatomic landmarks were defined in each set of images. A standard level set segmentation was applied to the training set. The level set segmentation was compared to the manual delineation for each of the cases in the training set. The training set images were aligned to each other by the use of the four landmarks. The spatial information on where to restrict the segmentation, called a correction map, was calculated as the mean of the difference between the level set segmentations and the manual delineations for the training set. The correction map and intensity information from the training set were stored into a prototype. The remaining five cases were used as test set and the segmentation was done by the use of the prototype and a level set method. In the test set the segmentation error was calculated as both a volumetric error and a mean absolute distance between the manual delineation and the prototype-based segmentation.

## Results

**Figure 1:**
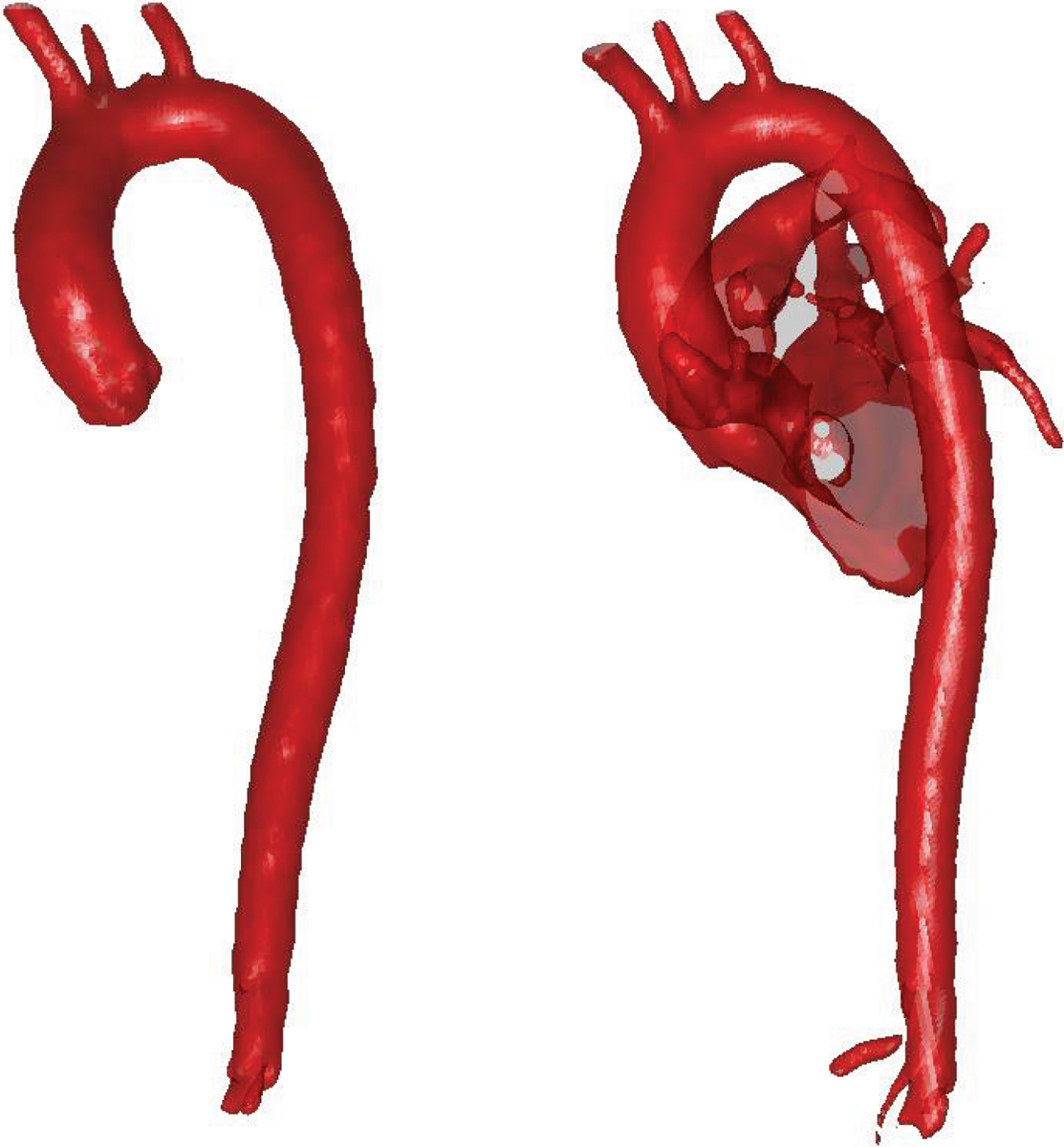
Figure 1

The segmentation error in the test set was (mean ± SD) 4 ± 5% when measured as volumetric error and 0.58 ± 0.06 mm when measured as the error in mean absolute distance. Figure 1 shows 3D surface rendering of the results of the prototype-based segmentation (left) compared to the results of a purely intensity based level set segmentation (right).

## Conclusion

The proposed segmentation algorithm is highly accurate, in particular considering the segmentation error expressed as mean absolute distance, which is on a sub pixel level. In the left panel of the figure it can clearly be seen that the prototype-based segmentation successfully constrains the segmentation to only include the aorta which can be compared to the purely intensity based segmentation in the right panel which includes part of the blood volume in the heart and in the pulmonary vessels. The greatest advantage with the prototype-based segmentation method is that only a small number of cases are needed to extract the necessary a priori information. Since only about five cases need to be manually segmented, a new prototype can easily be constructed when new MR pulse sequences are developed or new research fields arise. Future work includes applying the prototype-based segmentation to renal and carotid arteries.

